# Measurement of renal cortical fibrosis by CT scan

**DOI:** 10.1016/j.redii.2023.100024

**Published:** 2023-03-01

**Authors:** John D Olson, Janet A Tooze, Daniel J Bourland, J Mark Cline, Eduardo B Faria, Eric P Cohen

**Affiliations:** a Department of Comparative Medicine, Wake Forest University, Winston-Salem, NC, 27101, USA; b Department of Radiation Oncology, University of Maryland School of Medicine, Baltimore, MD, 21201, USA; c Nephrology Division, Department of Medicine, New York University Grossman School of Medicine, New York, NY, 10016, USA

**Keywords:** Renal cortical fibrosis, CT scan, Non-human primate

## Abstract

**Rationale and objectives:**

The accurate, non-invasive, and rapid measurement of renal cortical fibrosis is needed for well-defined benchmarks of permanent injury and for use of anti-fibrotic agents. It is also needed for non-invasive and rapid assessment of the chronicity of human renal diseases.

**Materials and methods:**

We have used a non-human primate model of radiation nephropathy to develop a novel method of size-corrected CT imaging to quantify renal cortical fibrosis.

**Results:**

Our method has an area under the receiver operating curve of 0.96, which is superior to any other non-invasive method of measuring renal fibrosis.

**Conclusion:**

Our method is suitable for immediate translation to human clinical renal diseases.

## 1. Introduction

Fibrosis is a marker, mechanism, and endpoint of chronic kidney disease [1–3]. Its accurate non-invasive measurement is thus of substantial interest. It was recently shown that size-adjusted CT scanning can quantify renal cortical fibrosis in non-human primates (NHP) [4]. That paper included only 10 NHP that had undergone total body irradiation and had renal fibrosis as a result. Confirmation of those findings is needed. In addition, the initial report used a single type of CT scanner; extension of our method to include different CT scanners is essential to enable its wider use. We report herein the confirmation of those findings in a larger cohort and the development of a method to correct for use of different CT scan machines.

## 2. Methods

A colony of previously irradiated rhesus macaque (*Macaca mulatta*) non-human primates (NHP) is maintained at our center. These animals were irradiated at different institutions for prior acute radiation studies. Then, animals were transferred to us to study the longer-term delayed effects of radiation exposure. All animal procedures were performed in accordance with the Animal Welfare Act (Animal Welfare Assurance Number A-3391–01) and the Guide for the Care and Use of Laboratory Animals (National Research Council. 2011). The ARRIVE guidelines were followed. Daily assessments included activity, fecal output, signs of edema, vomiting, hemorrhage, seizures, respiratory distress, and food intake. These assessments were performed a minimum of four hours apart and daily on weekends. Animals were fed a diet that mimics the North American Diet (5LOP, LabDiet, St. Louis, MO) with the addition of fruit, vegetables, and daily enrichments.

Animals underwent euthanasia if they: 1) had likelihood of unplanned death due to illness; 2) had abnormal behavior impacting the animal’s well-being; or 3) were selected for euthanasia based on peer-reviewed scientific priorities such as tissue collection. Euthanasia was achieved by anesthetizing the animal to a deep surgical plane of anesthesia with sodium pentobarbital (20 to 30 mg/kg i.v.) then exsanguination by perfusion via the left ventricle with 1 to 2 liters of cold NaCl or lactate Ringer solution.

Thirty-three animals were included, 21 NHP that had received 5 to 8 Gy total body irradiation 1 to 13 years previously, 2 age matched non-irradiated controls, and 10 NHPs that received 10 Gy chest only irradiation that had minimal renal radiation exposure. These ten have been analyzed for other studies [5]. As reported therein, the kidneys of chest-only-irradiated NHP received between 1% and 5% of the 10 Gy chest-only dose, i.e. between 0.1 and 0.5 Gy.

These 33 animals were selected because each animal had a single full body computerized tomography (CT) scan, without the use of intra-venous contrast, within seven months before death and had one or both kidneys preserved for histology.

CT imaging was performed on anesthetized animals using one of three CT scanners: 1) from 2012 to 2017 on a 32 slice Toshiba Aquilion Scanner (Tustin, CA, USA) at 120 kV, 300 mA, field of view (FOV) = 320 mm, matrix = 512×512, and slice thickness of 0.5 mm, 2) in 2018 on a 16 slice GE BrightSpeed Mobile Scanner (Boston, MA, USA) with the same scan voltage, current, and in-plane geometry, but with a slice thickness of 0.62 mm, or 3) from 2019 to the present on a Siemens SOMATOM Definition Flash CT Scanner (Munich, Germany) at 120 kV, 216 mA, FOV = 320 mm, matrix 512×512, and slice thickness of 0.5 mm. All scanners had monthly quality assurance performed to confirm the stability of the CT number for water at 0 +/− 3 HU.

**Fig. 1.**
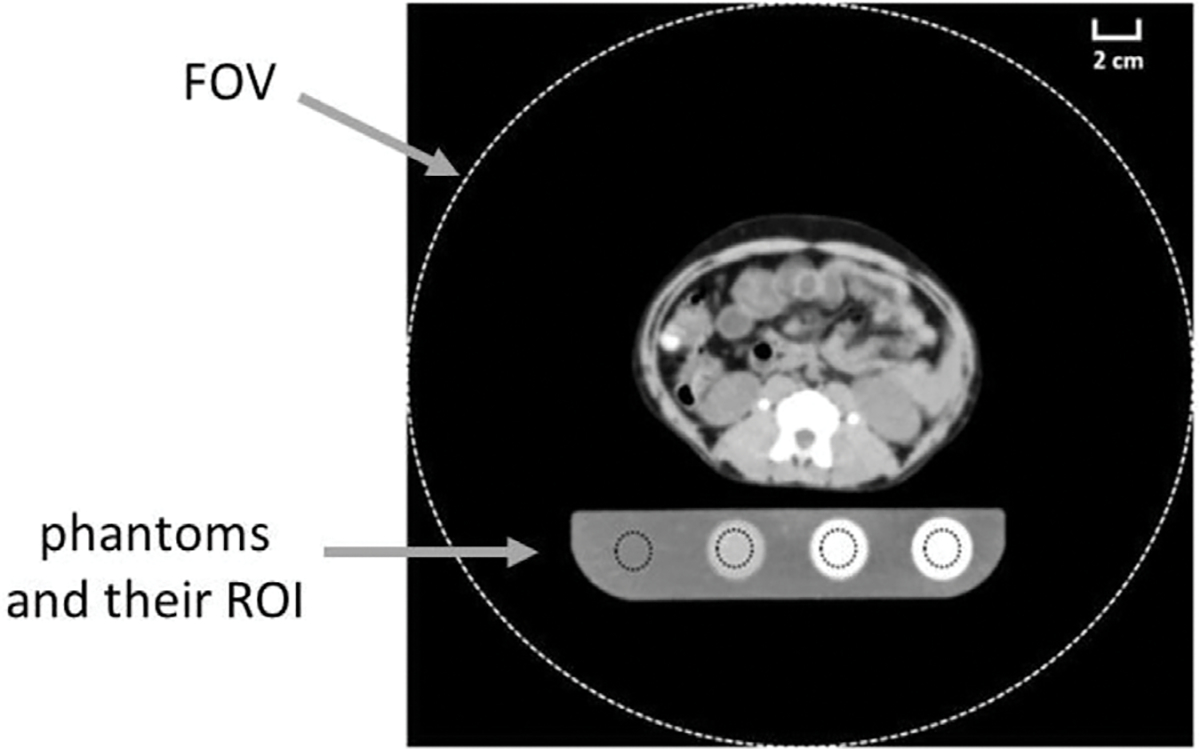
This shows a round total field of view (FOV) that was used to calculate the total size index (TSI). The in-field phantoms are also shown, with the region of interest (ROI) drawn for each.

Twenty of these 33 animals were used in a phantom-calibrated study to characterize each scanner’s residual beam hardening caused by size differences. These animals had the calibration phantoms in the scan FOV at the level of the torso (Fig. 1).

The scans did not include the trough used for positioning, because it created streak artifacts in the image of the phantom. The phantoms are four tissue neutral quantitative CT (QCT) cylinders that have 0, 50, 100, and 200 mg/ml of calcium hydroxyapatite. (Image Analysis, Inc, Columbia, KY, USA). Circular regions of interest (ROI) were drawn in the four phantoms using TeraRecon image analysis software (Durham, NC, USA) (Fig. 1).

The index of animal size was the total CT signal in the axial slice and was calculated by tracing a ROI that contained the entire field of view (FOV). The total CT signal in a slice was defined as the mean CT number for the entire FOV times the total area of the FOV. We subtracted the CT number for air (−1024 HU) from the mean CT number for the entire FOV. This ensured that the total size index (TSI) would be a positive number. That number was multiplied times the total area of the FOV in mm^2^. This value was divided by 10^7^ to yield TSI values between 0 and 10. The CT numbers of the phantom ROIs were plotted as a function of each size index. The relationship between CT number and subject size for each scanner was modeled by regressing the CT numbers for each phantom on the natural log of the TSIs. This had the form of y=A*Ln(TSI). The coefficient A was the average of the coefficients for the phantoms with densities just above and just below that of kidneys. These equations were used to correct for beam hardening caused by body size differences.

### 2.1. Scanner harmonization method

38 additional NHP were identified that were not part of the cohorts as identified above. These included NHP that were still alive, and also NHP whose CT body imaging was done over 210 days before their euthanasia. Each of these NHP were scanned on each of the three scanners used for this study: Toshiba, GE, and Siemens. To enable harmonization of CTHU readings, 9 of these 38 NHP were removed from this group because a trough was in the FOV or because their body weights at the time of the GE or Siemens scans differed by more than 10% of their body weight at the time of the Toshiba scan. The phantom HU were obtained as described above. The lowest density two phantoms (P1 and P2) were used for the harmonization because their HU are at the upper and lower limits of renal HU. This was done by calculating the difference between the HU of each of these two phantoms as measured by the GE and the Siemens scanners compared to the HU as measured by the Toshiba scanner. The coronal reconstruction of slices in the phantoms scanned with the GE mobile scanner showed a streak and dark band artifact. To minimize the effect of the artifact on the phantom ROI mean CT value, care was taken to have the phantom ROI span from mid maximum to mid maximum or mid minimum to mid minimum of adjacent maximums or minimums to standardize the ratio of bright voxels to dark voxels in the ROI. This was so that the bright or dark slices did not dominate the ROI, which thus included equal amounts of decreased and increased HU to have a minimal effect on the mean HU value for the ROI.

**Fig. 2.**
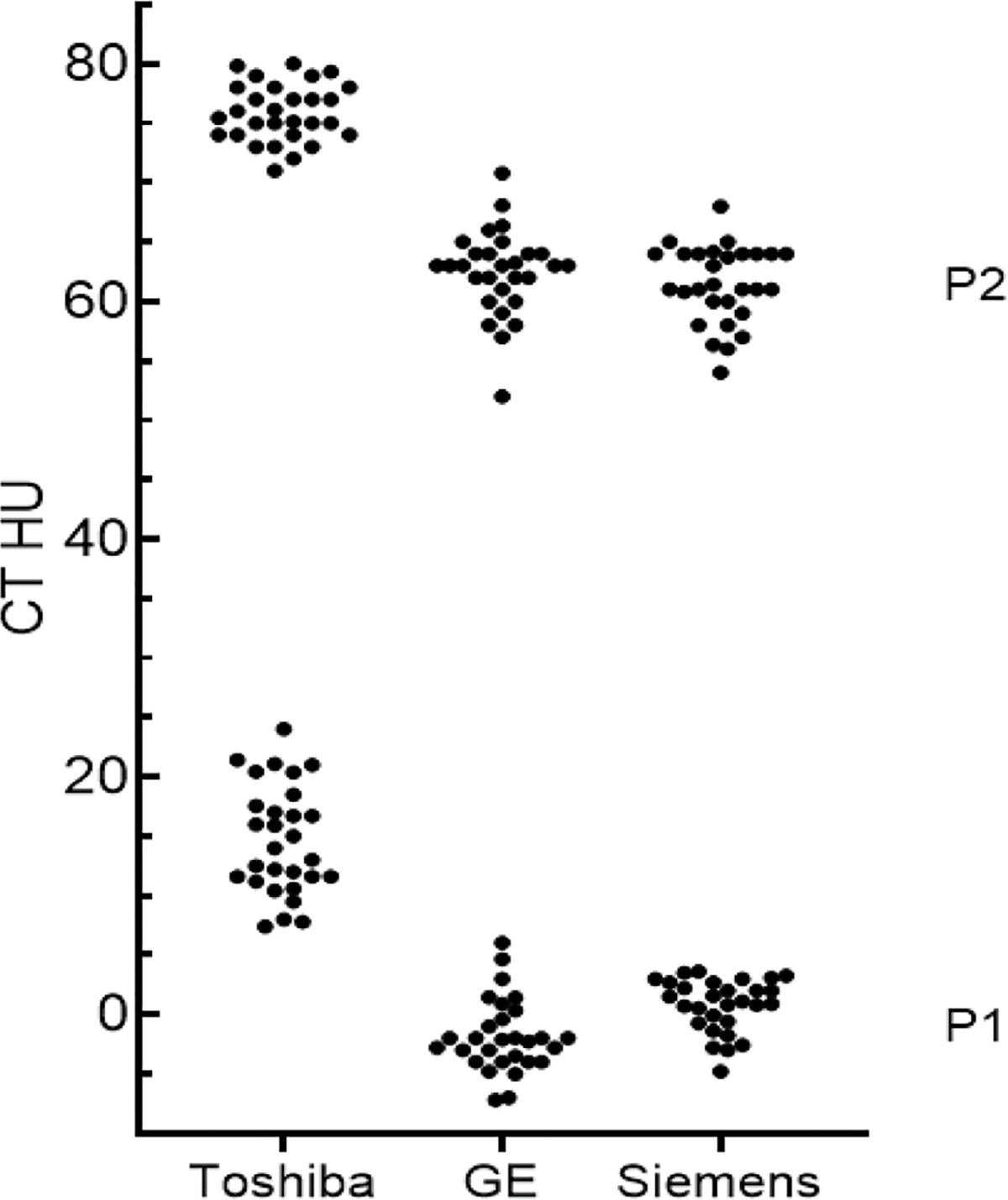
This shows the raw CTHU for phantoms 1 and 2 of the non-irradiated same-weight cohort that enabled the harmonization process. Each NHP was scanned on each of the three CT scanners. The average difference of the phantom HU values was used to harmonize the raw kidney CTHU obtained from the GE and Siemens scanners to the kidney HU of obtained using the Toshiba scanner.

Fig. 2 shows the raw values for the CTHU of the P1 and P2 phantoms for each NHP that was scanned on all three scanners.

The average differences of the phantom values between scanners were averaged to yield a number that harmonizes the “raw” HU values for the GE and Siemens scanners to those of the Toshiba scanner. Those numbers were used to harmonize the renal HU values that were obtained as described below.

**Fig. 3.**
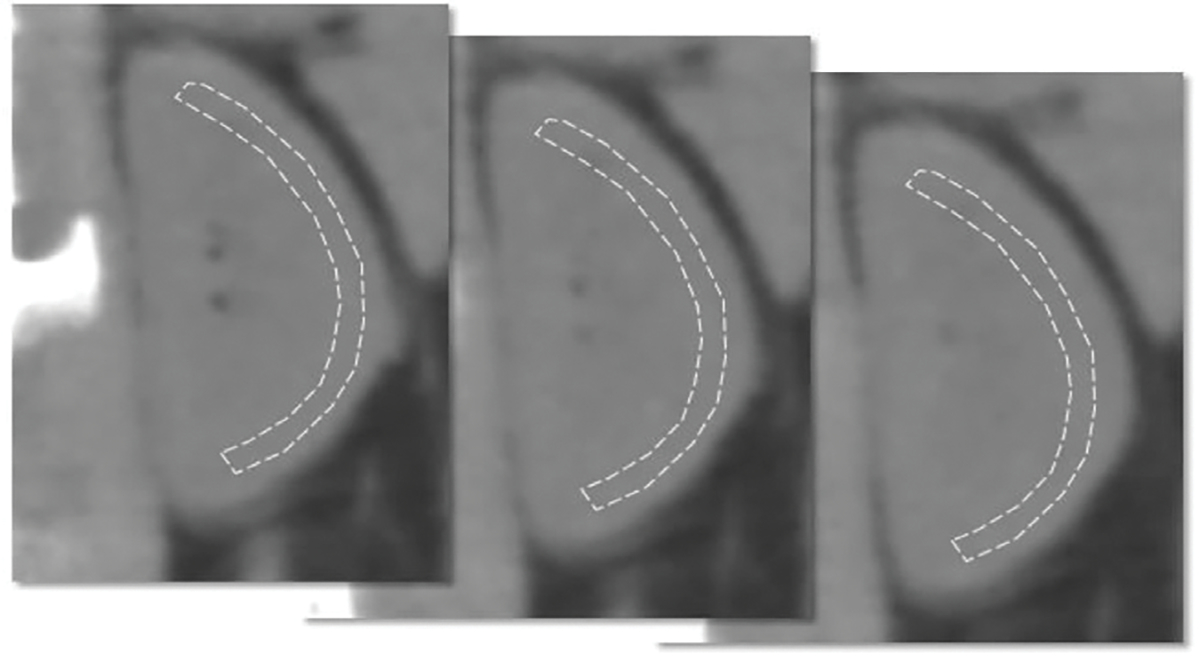
Three renal cortex ROIs are shown. The average of the three renal cortex ROIs was used as the raw CTHU for that kidney. The ROIs excluded medulla. They did not include cystic areas. There was no case showing hydronephrosis.

CT images were analyzed by a single researcher who was masked to the fibrosis score of the kidneys. Renal cortex ROIs were hand traced in three coronal slices and averaged to get a mean CTHU for that kidney’s cortex (Fig. 3).

The TSI for each kidney was calculated using three axial slices that intersected the renal cortex ROIs. The TSI of these three measurements were averaged to yield the TSI for each kidney. Each kidney’s CTHU was harmonized to the Toshiba scanner then its TSI was used to correct for body size.

Blood samples were obtained just before euthanasia and tested for blood urea and serum creatinine using commercial kits. The normal range of serum urea in NHP is 6.6 to 8.5 mmol/L and that of the serum creatinine is 53 to 80 micromol/L. Kidneys were formalin-fixed, paraffin embedded and sliced into 4 *μ*m thick whole-organ sections, two to four per animal. Sections were stained with Masson Trichrome.

**Fig. 4.**
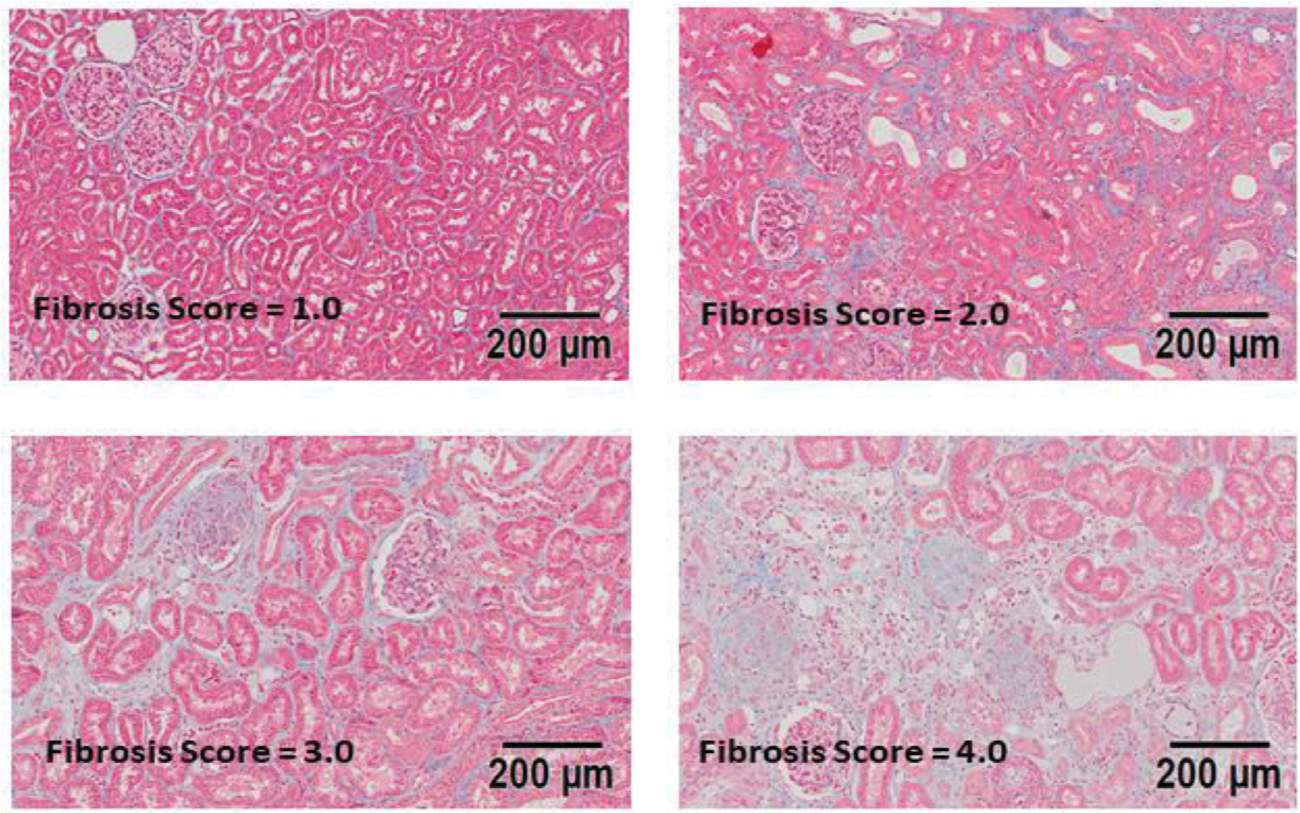
Representative histology of the four grades of renal cortical fibrosis, showing increasing extent of fibrosis from grade 1 to grade 4.

Cortical fibrosis was scored twice for each kidney in a masked fashion, as previously reported [4], on a scale of 0 through 4, by assessment of at least four fields at 100x magnification (0=none, 1=minimal, 2=less than half of fields fibrotic, 3=more than half of fields fibrotic, 4=all fields fibrotic) and the results averaged for each kidney (Fig. 4).

Method “B” as reported in Farris et al. [6] was used for assessment of tissue fibrosis, which is the assessment of tissue area that is scarred by fibrosis. Four NHP had kidney sections that were not identified as right or left; for those animals one of the kidney scans was chosen at random for the CT comparison. One NHP had a single kidney.

Renal cortical fibrosis was also measured by ImageJ, using the same tissue sections. The tissue area that stained blue was recorded, which corresponds to method “A” as reported in Farris et al. [6].

### 2.2. Statistical analysis

**Fig. 5.**
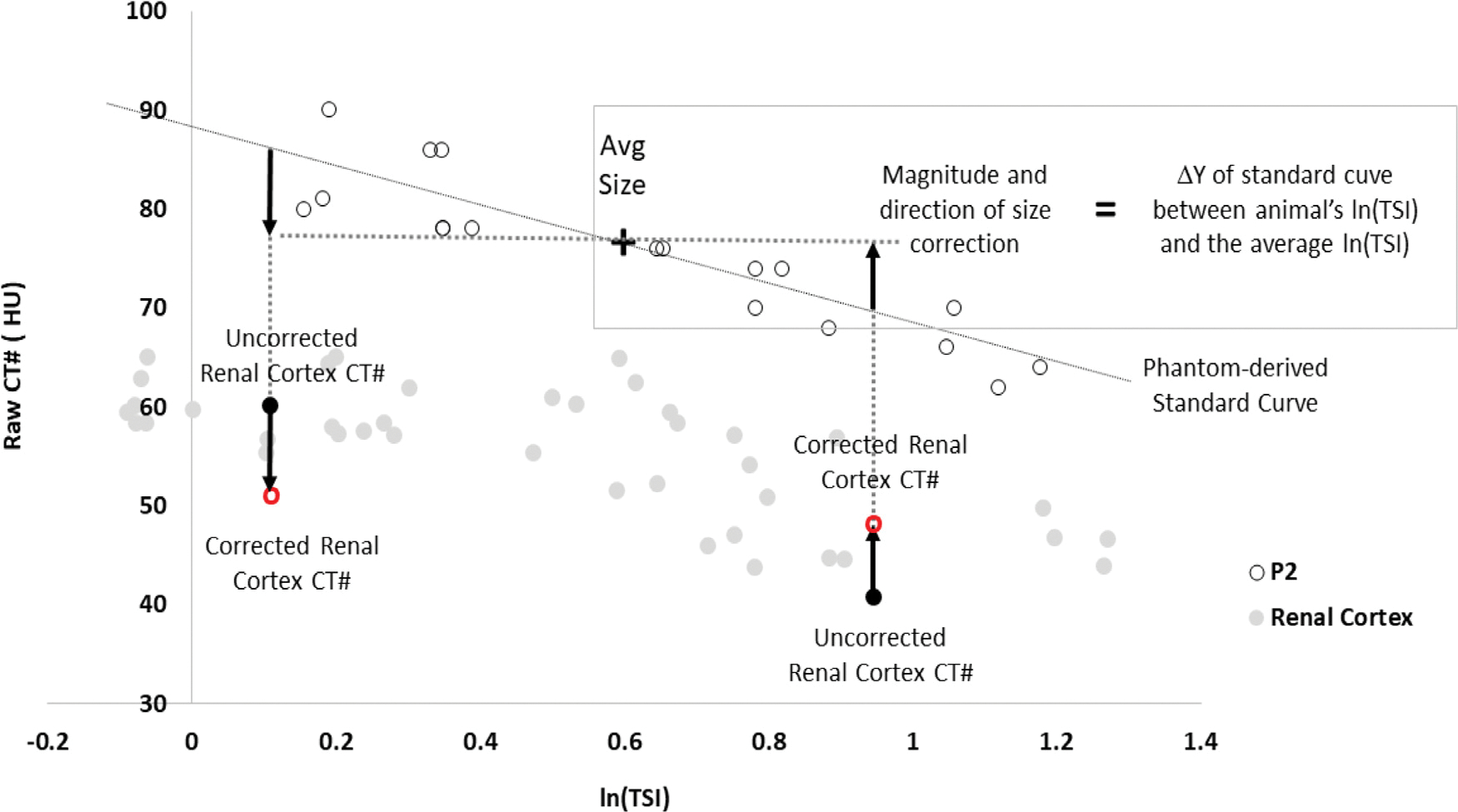
A graphical portrayal of the size correction process. The un-corrected “raw” CTHU correspond to phantom HU values on the standard curve. The increment of those values to the HU for the average size defines the upward or downward “shift” of raw value to the size-averaged corrected CTHU value.

We regressed the CTHU of the phantoms to the natural log of the corresponding total size index. Because the CT numbers of the tissue of interest in this study all fell between the values of phantom regions 1 and 2, we examined whether the slopes differed using a Wald test of interaction. The slopes were not significantly different; therefore, one equation was fit for phantom ROIs 1 and 2. Before the size correction, the “raw’ CTHU for the kidneys were harmonized to the values of the Toshiba scanner. These harmonized CTHU were considered the measured CTHU for each NHP kidney. To size-correct each kidney’s CTHU, we calculated the corrected CT number using the equation:

$$\text{Corrected}\;\text{CT}\;\text{HU}=\text{Measured}\;\text{CT}\;{\text{HU}}_{\text{i}}\;\;+\text{A}\ln\;(\text{size}\;{\text{index}}_{\text{i}}/\text{mean}\;\text{size}\;\text{index})$$

where measured ${\text{CT}\;\text{HU}}_{\text{i}}$ and ${\text{index}}_{\text{i}}$ are the values measured for each kidney, A is the slope of the phantom CTHU versus natural log of the size index regression and mean size index is the mean size index for the study cohort. This is shown graphically in Fig. 5.

**Fig. 6.**
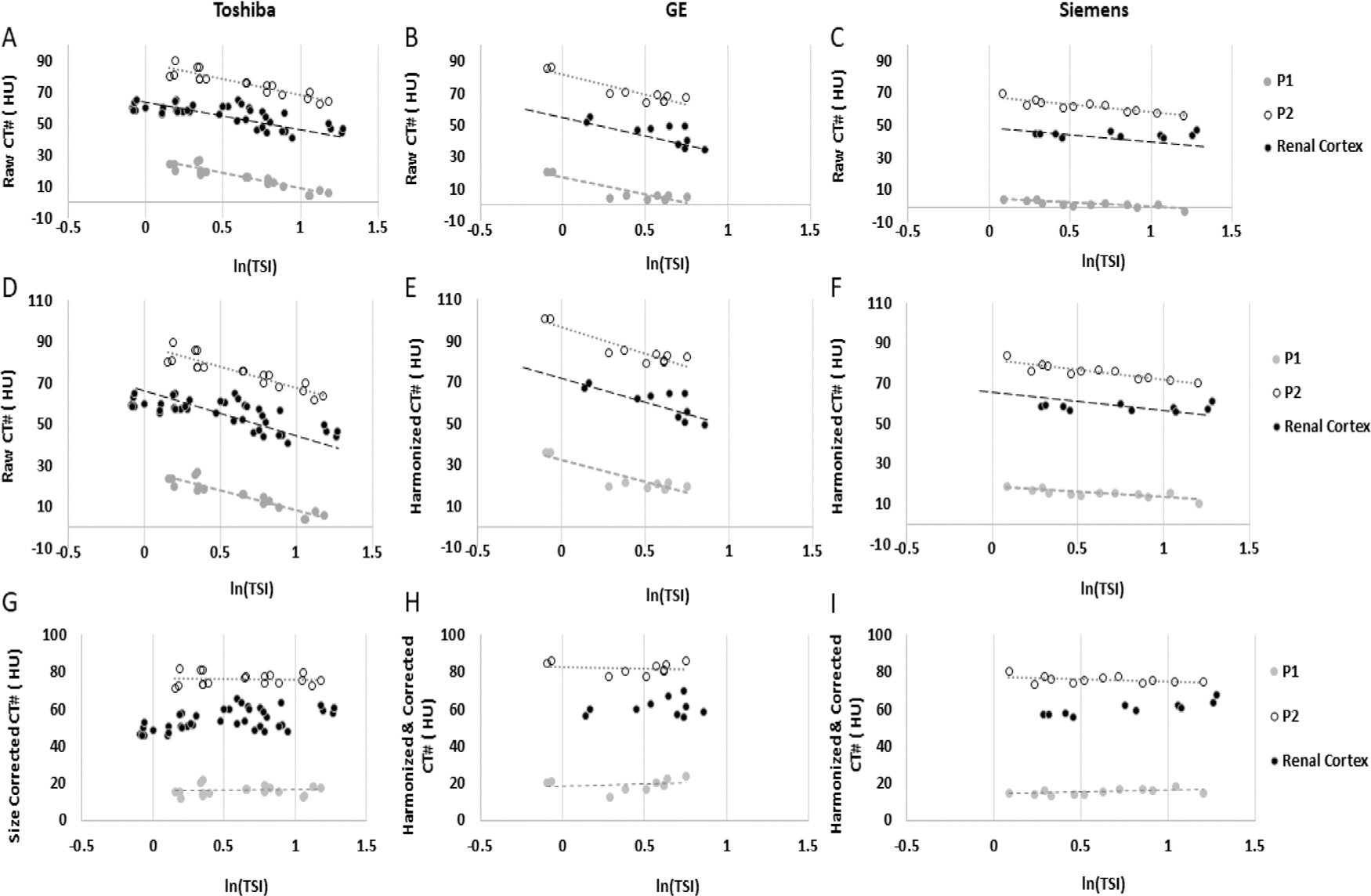
These graphs show the transformation from raw CTHUs (A-C) to CTHUs harmonized to one scanner (D-F) to harmonized and size corrected CTHUs (G-I). Top Row A-C: raw CTHUs vs ln(TSI) from P1 and P2 phantom ROIs and from the renal cortex ROIs from the three scanners used in this study. All three scanners exhibit the size dependent decrease in CTHU caused by beam hardening. The slope of the effect is scanner-dependent with the newest scanner, the Siemens having the lowest slope. Middle Row _D_-E: CTHUs harmonized to the Toshiba scanner vs ln(TSI). CTHUs from the GE and Siemens were increased by the mean difference between P1 and P2 values from a cohort of animals who were scanned on the three scanners. The Toshiba data did not need to be harmonized to match itself. Bottom Row G-I: Harmonized to Toshiba and size corrected P1, P2 and renal cortex CTHU values.

The combined harmonization and correction process is shown on Fig. 6.

Fibrosis was defined as a mean fibrosis score of 1 or greater. A generalized estimating equation (GEE) logistic regression model to account for repeated measures (kidney sides) on each animal [7], and receiver operating characteristic (ROC) analyses were performed to assess the ability of the corrected CTHU to discriminate fibrotic renal cortex from non-fibrotic renal cortex and to select a cut-off value that maximized sensitivity and specificity. To obtain an estimate of the correlation between methods (clinical scoring of histology, CT, histological fibrosis area using ImageJ, urea, and serum creatinine) accounting for the repeated measures by side, a mixed model with random effects was used, treating the method and the side as repeated measures on the animal and including method as a fixed effect to account for the different means by method for all animals, and for animals with fibrosis scores greater than zero.

## 3. Results

**Fig. 7.**
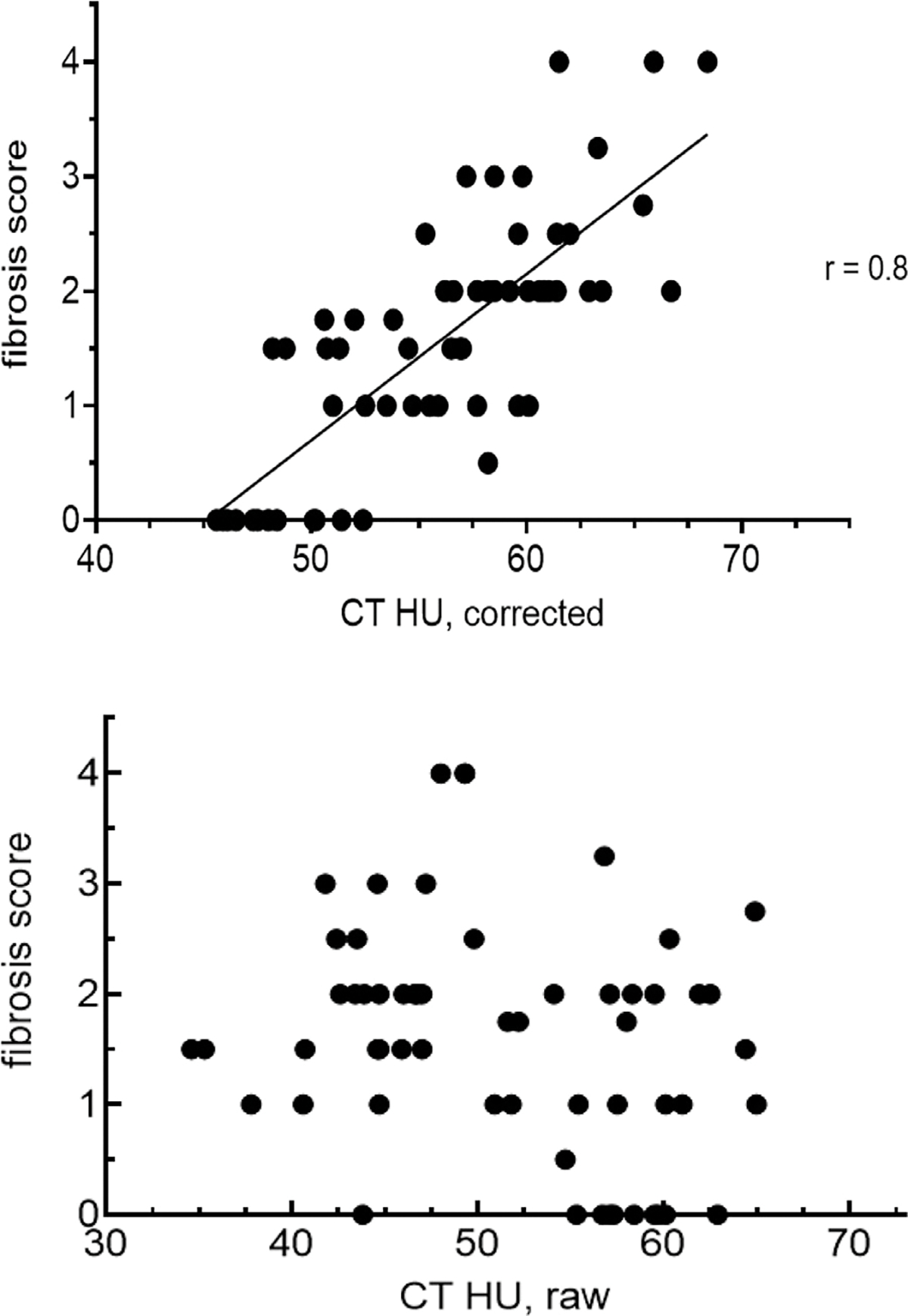
a. The relation of histological fibrosis score to the size-corrected CTHU. The excellent direct correlation is evident. A corrected CTHU of 52 or more corresponds to increased fibrosis in all cases. A corrected CTHU of over 60 corresponds to substantial renal cortical fibrosis. b. The relation of histological fibrosis score to the non-corrected CTHU. There is no correlation This shows the importance of the harmonization then the size-correction steps.

There was an excellent correlation between fibrosis score and size-corrected CT number (Fig. 7a, r=0.8, p<0.0001).

Evaluating only those kidneys with fibrosis > 0, the correlation between fibrosis score and corrected CT number was r=0.6 (p<0.0001). There was no correlation of the fibrosis score to the non-corrected CTHU (Fig. 7b, r=0.1, p=0.3).

**Fig. 8.**
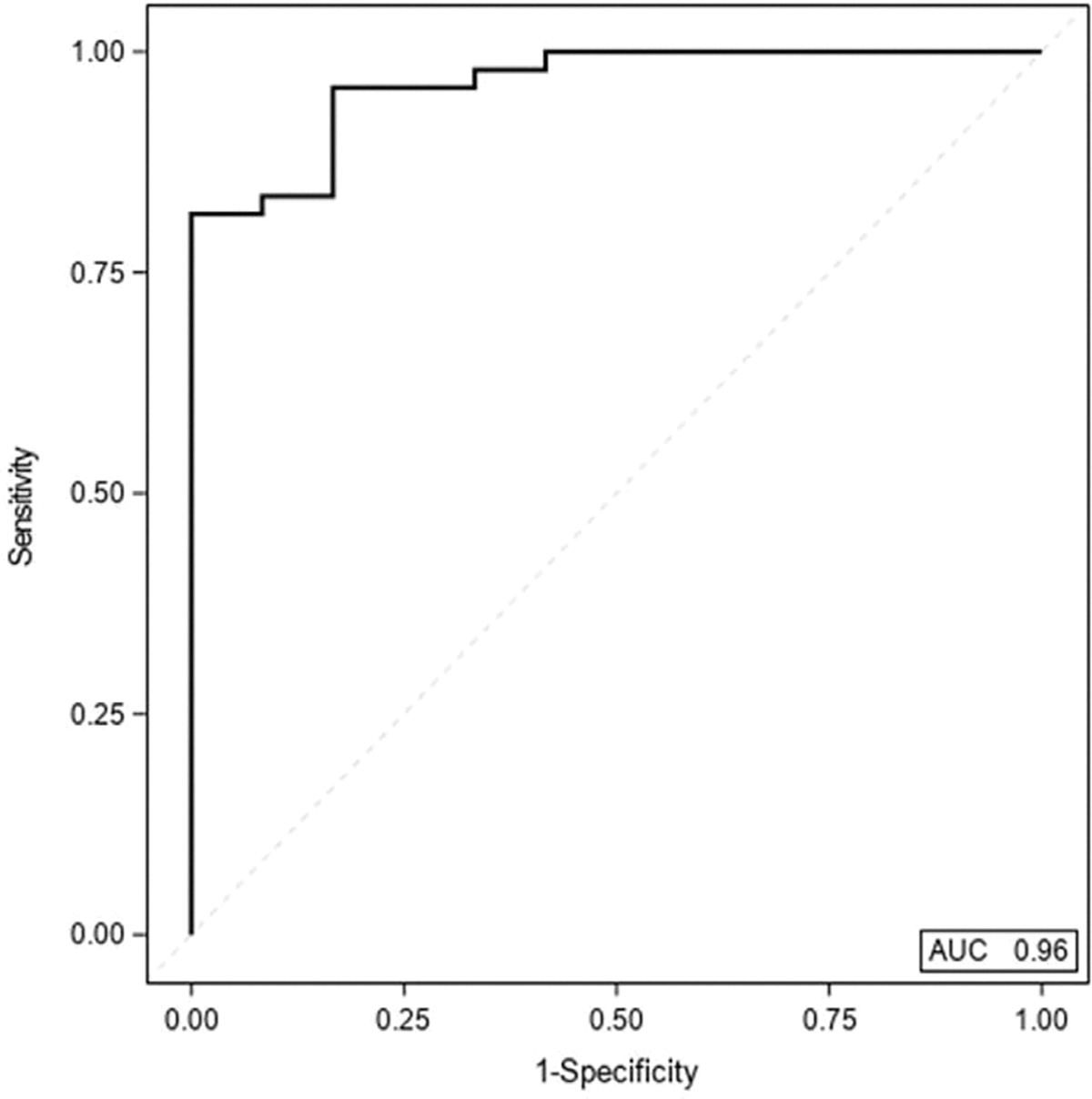
The area under the receiver operating curve for discrimination of renal cortical fibrosis from no-fibrosis using the size corrected CTHU of the renal cortex.

As shown on Fig. 8, the area under the receiver operating curve was 0.96, which indicates excellent discrimination of renal cortical fibrosis from no-fibrosis using the size corrected CTHU of the renal cortex.

**Fig. 9.**
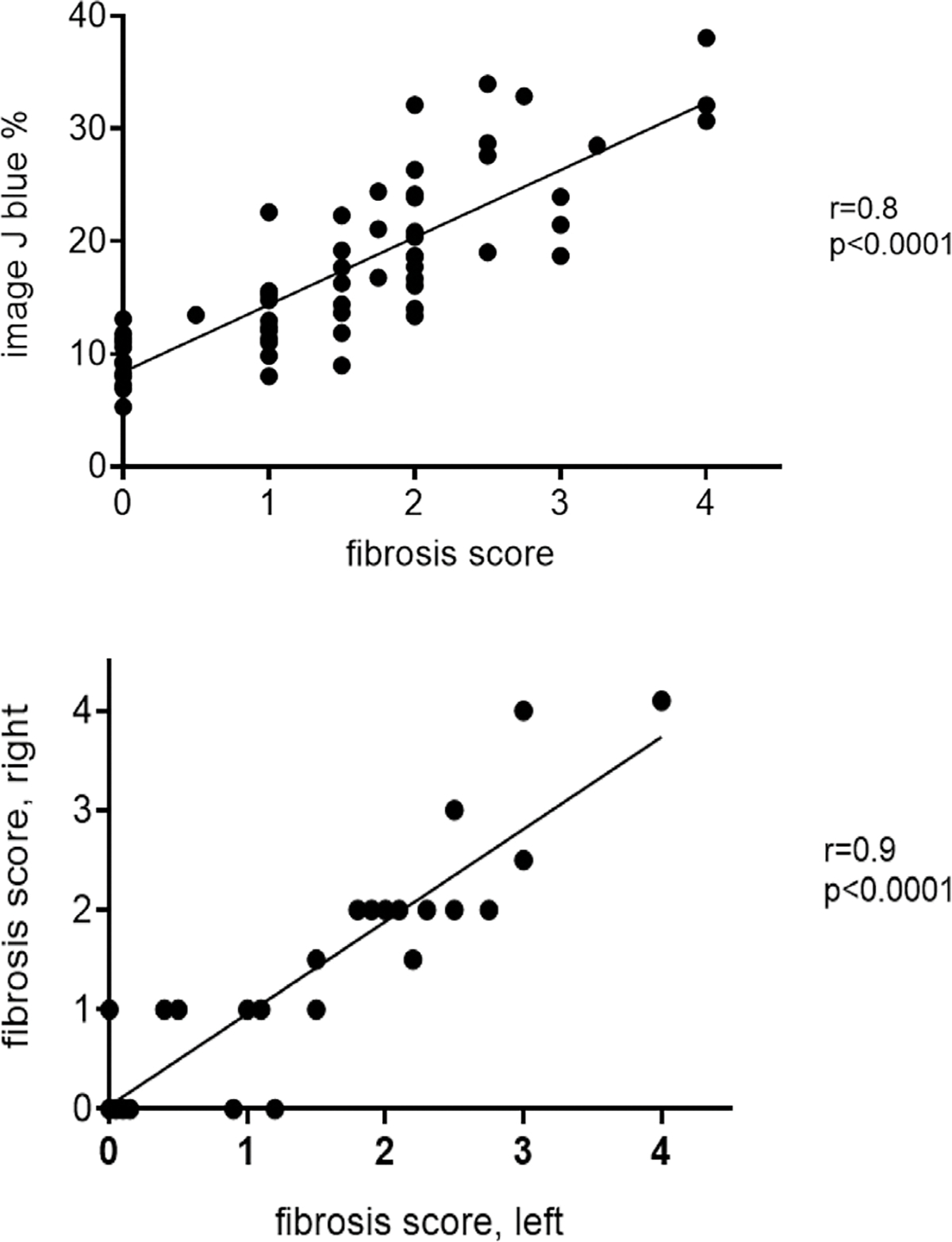
a. The relation of the histological fibrosis scores to the computer-assisted ImageJ scoring of tissue collagen in the same histological sections. There is an excellent direct correlation. b. The relation of the histological fibrosis score of the left kidney to that of the right. There is an excellent direct correlation, which confirms the reliability of the histological scoring method.

There was a very good correlation of the human scoring of fibrosis to that done by Image J (Fig. 9a, r=0.8,p<0.0001).

There was also excellent correlation of the human scoring of the left versus the right kidney of the NHP (Fig. 9b, r=0.9, p=<0.0001)

With an adjusted cutoff CT number value of 51 HU, the specificity was 83% and the sensitivity was 84%. The positive predictive value was calculated at 95%, and the negative predictive value was 56%. When the cutoff was below 48, the negative predictive value was100%; when the cutoff was above 52.4, the positive predictive value was 100%. The overall prevalence of fibrosis was 81%.

**Fig. 10.**
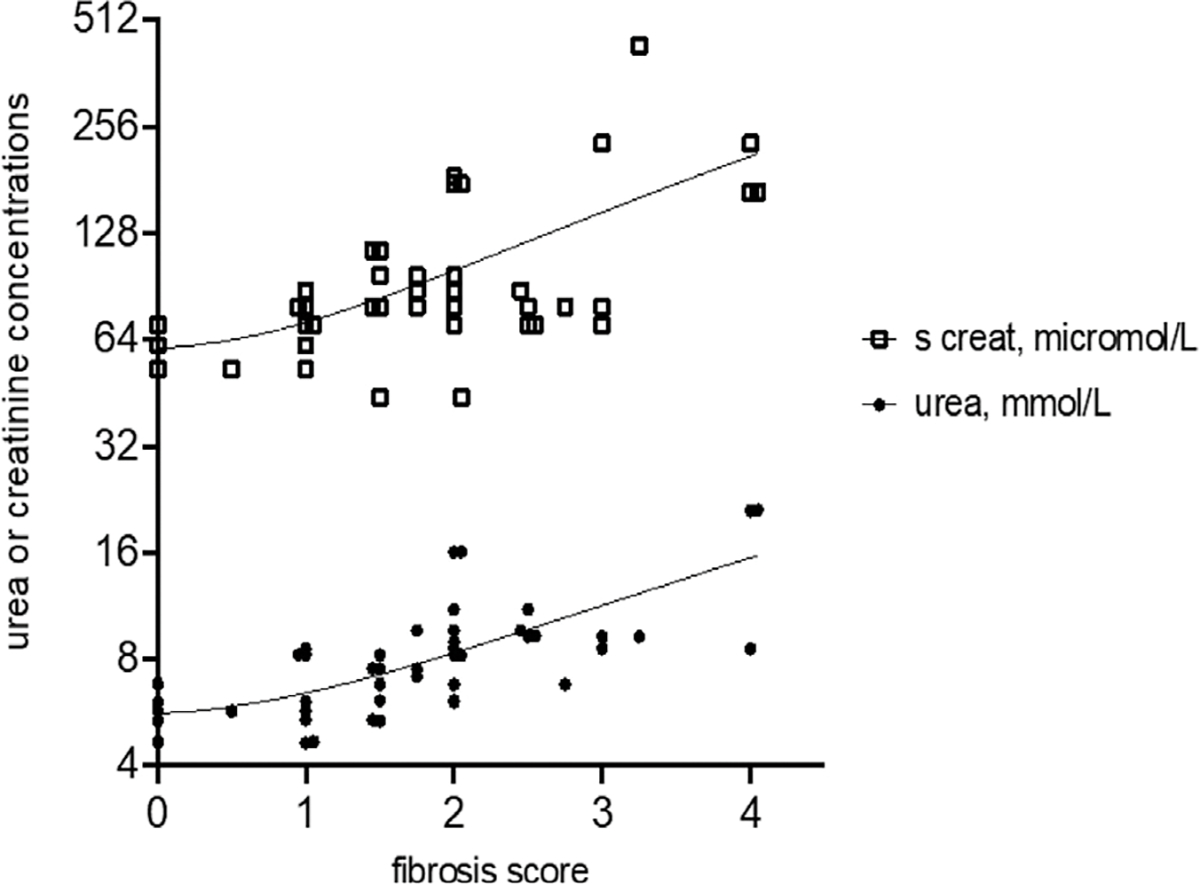
The relation of the histological fibrosis score to the serum urea and the serum creatinine. The best fit line is shown, and it is a second order polynomial. Some points have been nudged to avoid overlap. There appears to be some increase in tissue fibrosis before substantial azotemia, i.e. before there is marked loss of renal function.

There was a direct and significant correlation of the fibrosis score to the serum urea (r=0.7, p<0.0001) and creatinine (r=0.6, p=0.001) (Fig. 10).

## 4. Discussion

These data show a very good prediction of renal cortical fibrosis by use of size-adjusted CTHU of the renal cortex. We further confirm the correlation of semi-quantitative histological scoring to a computer-assisted method.

The direct correlation of BUN to histological fibrosis is consistent with studies that have correlated renal parenchymal scarring with renal function and with long term outcome of fibrotic kidney diseases [2,8]. In the present studies, it appears that the BUN and serum creatinine do not rise substantially until the fibrotic process is more than minimal, i.e. grade 2 or CTHU>55. This suggests that fibrosis begins before there is major change in renal function, and thus could be a mechanism that contributes to progressive renal failure, rather than just a marker [1,3,9].

The model used for these studies is of radiation nephropathy, a chronic fibrosing kidney disease that shows progressive collagen-rich fibrosis as do all chronic progressive kidney diseases. We expect that our findings apply to all chronic fibrosing kidney diseases.

The median time from CT to euthanasia in this cohort was 49 days. During this time, there could have been progression of parenchymal injury and fibrosis. Then if renal tissue had been obtained at the same time as the CT scan, the histology fibrosis scores relationship with the CTHU would be shifted. To address this possibility, we tested the relationship of histological fibrosis scores to CTHU in NHP that had their CT scan within a month of euthanasia. The correlation remained robust, with r=0.8.

Our fibrosis scoring method corresponds to current clinical practice. The amount of fibrosis in a renal biopsy is usually reported in a semi-quantitative way, from absent to scattered, to moderate, then extensive [8]. This supports the practicality of the correlation that we find between histological fibrosis and CTHU.

The excellent correlation of left and right kidney histological fibrosis scores is expected because of the uniformity of radiation dose delivery, and it confirms the consistency of the histological scoring.

### 4.1. Scanner harmonization

In addition to our method for size correction, we have developed a method to harmonize the HU readings of different CT scan machines. This emphasizes that use of our method to quantify fibrosis must take CT settings into account by use of phantom measurements to enable harmonization.

### 4.2. Competing methods

This report compares our CT method to the histological “gold standard” for fibrosis, and not to the many other non-invasive methods that attempt to identify renal fibrosis. Imaging methods that have been tested for that purpose include ultrasound and magnetic resonance imaging (MRI). Neither show very good correlations with renal fibrosis [10,11], and neither have the resolution of our CT method, which is at the level of 0.5 mm. In addition, ultrasound has the disadvantage of being in part dependent on the operator of the ultrasound equipment. MRI is more expensive, involves long scan times, and cannot be done in subjects who are claustrophobic or who have metal in their bodies. Further, MRI techniques depend on indirect tissue correlates of fibrosis, such as estimated water movement that is reported as the apparent diffusion coefficient, whereas our CT method depends directly on the increased tissue density that is caused by collagen. There is a recent report of the use of CT and an artificial intelligence method that identifies interstitial fibrosis by identifying patterns of relatively dense voxels. The AI method may differentiate its histological severity [12]. The success of the AI method supports our finding that the presence of fibrosis can increase the CT density of affected voxels enough to be detected in CT data, but this method currently depends on an algorithm that is not routinely available. Moreover, while this method differentiated severe from not-severe fibrosis, it did not show a graded relation between extent of fibrosis and the CT images. Urine tests can identify metabolites such as collagen degradation products or even mediators such as TGF-beta. But these are generally imprecise, not kidney-specific, and none have been identified in a non-human primate model [13–15].

### 4.3. Sampling, cost and availability

Renal biopsy remains the “gold standard” for assessment of renal fibrosis. But it is a small sample, having a surface area measured in square millimeters. The CT ROI measured in this study and that can be measured in humans has a surface area in the square centimeter range. That provides more information than does a biopsy or even multiple biopsy cores. One can estimate the cost of a renal biopsy at 3000 to 6000 US dollars. The cost of a non-contrast abdominal CT scan is from 1500 to 3000 US dollars. Renal biopsy requires preparation, is not safe in subjects who are on anticoagulants, and is not available at all medical centers. Non-contrast CT scanning requires little or no preparation, poses no risk to subjects on anticoagulants, and is widely available. Its result is also available immediately, whereas biopsy results often are only available after days.

### 4.4. Limitations

It is possible that the correlation of CTHU to renal cortical fibrosis shown in the present studies would only hold for radiation-induced fibrosis. Use of other models of renal fibrosis will eliminate that concern.

The “gold standard” for in-life assessment of renal cortical fibrosis is a renal biopsy. These have proven diagnostic value, and also enable prognosis based on the extent of fibrosis in the biopsy sample [2,8]. But renal biopsy is not a uniformly innocuous procedure. Hematuria may occur in 10% of cases, requiring transfusion or intervention in 1 in 10 of those. Uninephrectomy may ensue, in approximately 1 in 1000 cases. Death associated with renal biopsy has been reported in 1 in 1667 biopsies [16] and another report cites an alarming 1 in 100 occurrences of death associated with renal biopsy [17]. These actual data are in contrast to the un-proven risk of cancer caused by a CT scan.

## 5. Conclusions

The present studies are likely to be translatable to human use, and that is a goal of future studies. Once those confirm this method, its wider use can be applied to human clinical practice. But that translation from NHP to humans is first necessary. Then, our CT method could supplant the use of renal biopsy to assess renal fibrosis, enable its early and rapid identification, and also decrease the cost and improve the safety of studies that seek to identify or treat renal fibrosis.
